# Ferret acute lung injury model induced by repeated nebulized lipopolysaccharide administration

**DOI:** 10.14814/phy2.15400

**Published:** 2022-10-21

**Authors:** Oula Khoury, Cara Clouse, Malcolm K. McSwain, Jeffrey Applegate, Nancy D. Kock, Anthony Atala, Sean V. Murphy

**Affiliations:** ^1^ Wake Forest Institute for Regenerative Medicine Wake Forest University School of Medicine Winston‐Salem North Carolina USA; ^2^ Department of Clinical Sciences, College of Veterinary Medicine North Carolina State University Raleigh North Carolina USA; ^3^ Department of Pathology/Comparative Medicine Wake Forest University School of Medicine Winston‐Salem North Carolina USA

**Keywords:** acute lung injury, acute respiratory distress syndrome, ferret model, lipopolysaccharide, lung function testing

## Abstract

Inflammatory lung diseases affect millions of people worldwide. These diseases are caused by a number of factors such as pneumonia, sepsis, trauma, and inhalation of toxins. Pulmonary function testing (PFT) is a valuable functional methodology for better understanding mechanisms of lung disease, measuring disease progression, clinical diagnosis, and evaluating therapeutic interventions. Animal models of inflammatory lung diseases are needed that accurately recapitulate disease manifestations observed in human patients and provide an accurate prediction of clinical outcomes using clinically relevant pulmonary disease parameters. In this study, we evaluated a ferret lung inflammation model that closely represents multiple clinical manifestations of acute lung inflammation and injury observed in human patients. Lipopolysaccharide (LPS) from *Pseudomonas aeruginosa* was nebulized into ferrets for 7 repeated daily doses. Repeated exposure to nebulized LPS resulted in a restrictive pulmonary injury characterized using Buxco forced maneuver PFT system custom developed for ferrets. This is the first study to report repeated forced maneuver PFT in ferrets, establishing lung function measurements pre‐ and post‐injury in live animals. Bronchoalveolar lavage and histological analysis confirmed that LPS exposure elicited pulmonary neutrophilic inflammation and structural damage to the alveoli. We believe this ferret model of lung inflammation, with clinically relevant disease manifestations and parameters for functional evaluation, is a useful pre‐clinical model for understanding human inflammatory lung disease and for the evaluation of potential therapies.

## Graphical abstract

We developed a ferret model of lung inflammation, with clinically relevant disease manifestations and parameters for functional evaluation, which can be a useful pre‐clinical model for understanding human inflammatory lung disease and for evaluation of potential therapies.

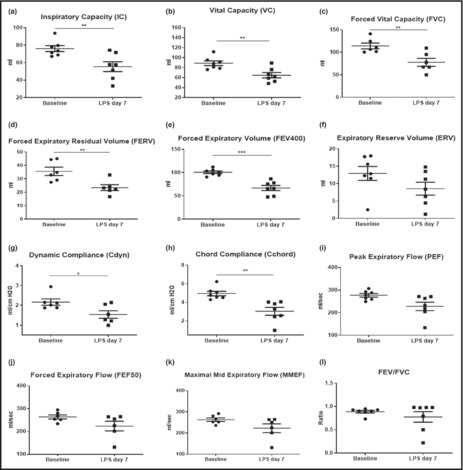

## 1 INTRODUCTION

Inflammatory lung diseases affect millions of people worldwide. Severe and unresolving inflammation in the lungs and airways has been associated with structural changes and damage to the pulmonary tissues and a decline in lung function (Crimi & Slutsky, 2004; Matthay et al., 2012, 2019; Meduri et al., 1995).

As many anti‐inflammatory therapies fall short in treating the persistent pulmonary inflammation in patients affected with these diseases (Araz, 2020; Yin & Bai, 2018), more studies are needed to understand the early mechanisms driving tissue injury, progression from early inflammatory injury to long‐term loss of pulmonary function, and to aid identification and testing of new therapies. Pre‐clinical animal models than recapitulate the severe and unresolving pulmonary inflammation, as well as the clinical loss of function, is needed. Rodents have been used to model various lung diseases, and have been extensively reported for providing important insights into the pathogenesis of human lung diseases such as acute respiratory disease syndrome (ARDS) and asthma (D'Alessio, 2018; Debeuf et al., 2016). However, relevant differences between human and mouse anatomy, physiology, and immunology, need to be considered when extrapolating findings from model to disease. (Bastarache & Blackwell, 2009; Bennett & Tenney, 1982; Gharib et al., 2010; Gomes & Bates, 2002; Irvin & Bates, 2003; Proudfoot et al., 2011; Wagers et al., 1985). Larger animals such as pigs, ferrets, and non‐human primates have been adopted to better model human lung disease as they have similar lung anatomy and biology to humans (Jung et al., 2009; Khatri et al., 2018; Liu et al., 2020; Miller et al., 2017; Rogers et al., 2008; Ryan et al., 2018). The use of a larger animal to model lung diseases has advantages, especially when performing pulmonary function testing (PFT). Larger animals have larger airways that permit easier and less invasive intubation with inflatable cuffs that form tight tracheal seals and prevent air leakage.

Rodent models have contributed valuable insights into human lung diseases such as acute respiratory distress syndrome (ARDS), asthma, and idiopathic pulmonary fibrosis, among others, however, their smaller size poses a challenge for PFTs (Aeffner et al., 2015; Marques‐Garcia & Marcos‐Vadillo, 2016; Mercer et al., 2015; Tashiro et al., 2017). Measurements such as compliance and airway resistance can be obtained in mice, however, procedures are invasive and often terminal. Other less invasive PFT approaches (Bates & Irvin, 1985; Bonnardel et al., 2019; Glaab et al., 1985, 2007; Glaab & Braun, 2021; Irvin & Bates, 2003) like plethysmography have been developed and are widely used in rodent studies to measure lung function in live conscious mice, thus eliminating the need for sedation, intubation, or tracheostomy (Hoymann, 2012; Quindry et al., 2016). However, because mice are spontaneously nose breathing during the procedure, the contribution of nasal passages and glottal aperture, in addition to the uncertainty of measuring bronchoconstriction are concerns that need to be considered, hence studies often combine these approaches with more invasive analyses (Glaab & Braun, 2021; Lim et al., 2014). Another method of measuring lung function is the use of the ex vivo lung perfusion approach which permits lung function measurements in isolated lungs. Although this approach overcomes the necessity and complications of intubation and sedation, it does not allow longitudinal studies and investigation of other physiological contributions that play a role in lung injury and disease progression as well as immune cell responses (Cardenes et al., 2021; Nelson et al., 2014; Wang et al., 2020). With the use of the ferret in this study, we were able to overcome these challenges and obtain repeated, precise, and reproducible lung function measurements with a minimally invasive approach.

Ferrets show high susceptibility to pathogenic infections, have increased total lung capacity, express known disease mutations, and show similar pathophysiology, tissue damage, and disease progression as human patients, making ferrets suitable models to study human viral infections (Wong et al., 2019), cystic fibrosis (CF) (Sun et al., 2010, 2014), and lung cancer (Aizawa et al., 2013), among others (Vinegar et al., 1985). Ferret lung physiology and the recent successes in the application of ferret models for various other pulmonary diseases suggest that the ferret may also be suitable as a pre‐clinical animal model for inflammatory lung injuries.

Inflammatory lung injury can be triggered by many factors. Bacterial antigens activate an inflammatory response characterized by increased infiltration of neutrophils, lymphocytes, and macrophages into the lungs, which are accompanied by elevated levels of pro‐inflammatory mediators including tumor necrosis factor (TNF)‐α, interleukin (IL)‐8, neutrophil elastase (NE), and reactive oxygen species that cause tissue damage to the lungs (Goodman et al., 2003; Grommes & Soehnlein, 2011; Han & Mallampalli, 2015; Lang et al., 2002; Sattar & Sharma, 2020). In this study, we induced inflammatory lung injury by administering lipopolysaccharide (LPS) into ferrets' lungs. The use of LPS to induce lung injury can closely mimic the clinical aspects of inflammatory lung disease including activated immune response, neutrophilia, and decreased lung function observed in human patients (Calama et al., 2017; Lei et al., 2018; Pera et al., 2011; Vernooy et al., 2002; Zhou et al., 2018). To create a targeted injury, we nebulized LPS to provide a direct delivery into the lungs. We also adopted a repeated exposure approach in which animals received nebulized LPS daily designed to mimic the natural history of human patients' continuous inflammatory response that leads to lung tissue damage and functional decline (Calfee et al., 2014; Domscheit et al., 2020; Patel et al., 2012; Song et al., 2019; Verjans et al., 2013). Thus, by exposing ferrets to LPS daily, we would maintain an ongoing response and potentially recapitulate the downstream lung tissue damage (Kaneko et al., 2007).

Following an insult to the lung, the change in lung function constitutes one of the most important indicators of the severity of lung injury and provides important parameters for diagnosis and treatment options in the clinic. Hence, we performed pulmonary function testing (PFTs) in the ferrets to evaluate lung function changes following LPS exposure. We performed repeated forced maneuver PFTs and established lung function parameters at baseline before the administration of LPS. In our current study, PFT procedures were only performed twice per animal, we have demonstrated that this can be performed multiple times per animal, with no detrimental effects from the intubation or forced maneuvers PFT procedure. This advantage will be most beneficial for long‐term longitudinal studies, such as evaluation of PFT changes in inflammatory lung injury caused by different factors (eg. chemical, viral, microbial) as well as to better understand how early PFT parameters may serve as useful predictors of long‐term outcomes. This is the first study to report the use of forced maneuver PFT in ferrets, establish lung function parameters at baseline in healthy ferrets and report changes in lung function post LPS injury. This animal model, with repeated daily doses of nebulized LPS in ferrets, closely represents multiple pulmonary manifestations associated with lung injuries, such as pulmonary neutrophilia, alveolar structure distortion, and decline in lung function, and thus provides a useful pre‐clinical platform for therapeutic and mechanistic studies of ALI. The repeated PFT performed on ferrets represents clinically relevant findings and permitted a more accurate evaluation of the LPS‐induced decline in lung function.

In addition, this model could be useful for the evaluation of respiratory diseases and treatments, such as pneumonia and Covid‐19‐associated ARDS, as it shares similar disease outcomes, associated inflammation, and injury with human patients that are affected by these diseases (Hu et al., 2020).

## 2 MATERIALS AND METHODS

### 2.1 Animals

**FIGURE 1:**
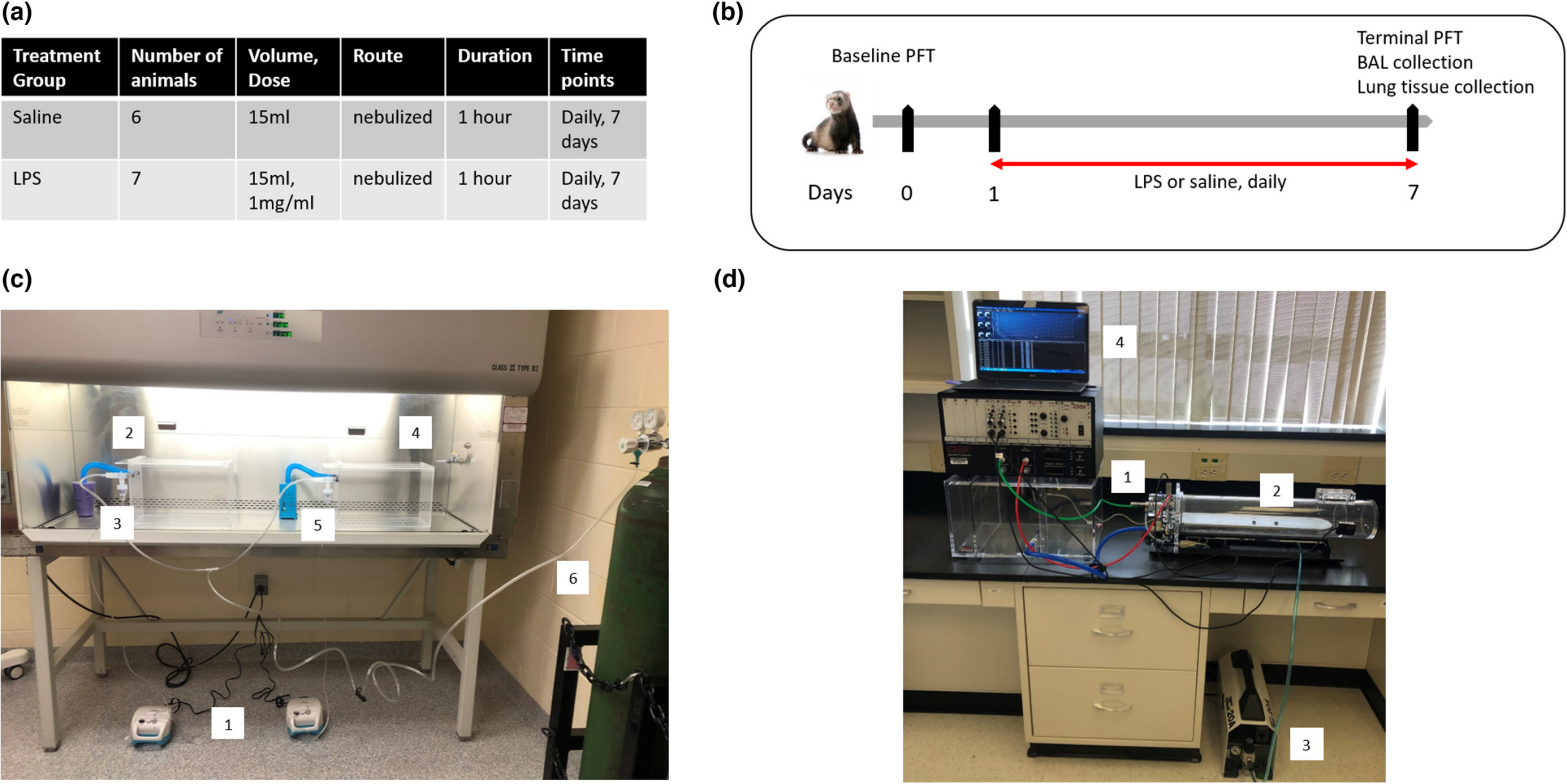
Study design. (a) Table summarizing the ferret groups used. (b) Study diagram and timeline. (c) LPS treatment setup: 1. Allied Schuco® S5000 Nebulizer, 2. Saline chamber connected to canister, 3. Saline cup connected to nebulizer and saline chamber. 4. LPS chamber connected to canister. 5. LPS cup connected to nebulizer and LPS chamber, 6. O_2_ tank. (d) Complete system integration: 1. The Buxco® Pulmonary Pressure Panel unit and chamber, 2. Exposure animal chamber, 3. Silentaire Super Silent 20‐A Air Compressor, 4. Personal laptop housing the Buxco FinePointe Software.

Male, neutered sable ferrets were purchased from Marshall BioResources, and were used under institutionally approved IACUC protocol A17‐078. Each animal weighed, on average, 0.8 kg and was 3–4 months old. They fasted approximately 3–4 h prior to any anesthetic events. A total of six animals received saline and seven animals received LPS (Figure 1a).

### 2.2 LPS administration

LPS derived from *Pseudomonas aeruginosa* (Sigma) was solubilized in 0.9% sodium chloride solution (Baxter) to a final concentration of 1 mg/ml. A total of 7 doses (1 mg/ml, 15 ml each) were administered to ferrets via a nebulizer, each dose delivered for 1 h daily for 7 days (Figure 1b). LPS was delivered to conscious ferrets using Allied Schuco® S5000 Nebulizer (Allied) in a tightly sealed chamber supplied with O_2,_ (4 L/min) (Figure 1c). Daily animal monitoring for adverse events included observations of food/water intake, bowel movement, animal activity, and signs of pain.

A total of 15 ml of saline was administered to ferrets via a nebulizer in control groups. Lung function measurements were performed at baseline and day 7 post LPS or saline delivery. BAL samples were collected on day 7 post LPS or saline administration. Baseline PFTs were performed 1 day before the first exposure to LPS.

Ferrets were closely monitored pre‐, intra‐, and post‐nebulization for any signs of erythema/swelling of the sclera and conjunctiva. We also watched for tearing and blepharospasm (blinking/winking). None of these signs were observed in any animal. We used a damp towel to wipe down the pelage of each animal post‐nebulization with the intent to minimize skin exposure. This also mitigated the risk of the animal getting LPS from its coat onto its mucous membranes as well.

### 2.3 Anesthesia for pulmonary function testing

The anesthesia that was selected for the ferrets was optimized to provide a balance between minimizing cardiopulmonary depression and providing appropriate planes of sedation required for forced pulmonary maneuvers via mechanical ventilation (the PFT apparatus). The drugs themselves were a combination of a muscle relaxant (~1 mg/kg xylazine), dissociative (~40 mg/kg ketamine), and a micro‐dose of opioids (~0.003 mg/kg buprenorphine). The xylazine and buprenorphine were on board solely to counteract hypertension and muscle tensing generally caused by ketamine. Additionally, the actual dosing of the sedation cocktail was performed gradually toward the maximums described above, and according to vital sign parameter thresholds rather than absolute dosing regimens. Ferrets were slowly given the smallest dose IV until the heart rate decreased to a certain period. Each animal was not placed on the PFT apparatus until the heart rate had normalized. In the event that any respiratory compromise was observed (e.g. during intubation), the animal was allowed to recover to the point of continuous spontaneous respirations prior to being fitted onto the PFT machine. This level of sedation consistently provided us with minimal sedation and cardiopulmonary dyscrasia. Additionally, this cocktail and its dosing procedure allowed the ferrets to have sufficient muscle relaxation and reduction in distress such that motion artifacts (e.g., trembling and “fighting the ventilator”) were negligible.

### 2.4 Pulmonary function testing

Lung function was assessed using the Forced Pulmonary Maneuver System (Buxco Electronics, Inc.) (Figure 1d). Briefly, ferrets were anesthetized using the cocktail described in the supplemental methods, intubated and placed in the system's body chamber. PFTs were performed using the FinePointe software. A series of testing optimization was performed on live ferrets, and parameters were set for our study according to Table 1.

**TABLE 1:** PFT parameters set for the study

Parameter	Settings
Total Lung Capacity (TLC) range	50–150 ml
FRC flow range	±8 ml/s
Normal flow for the PV test	5× FRC flow
High flow for the FV test	8× Normal Flow
Deadspace	1.25 ml
Positive and negative pressures	±60 cm H_2_O
Inflation rate	38 ml/s
Slow expiration rate	–23 ml/s
Flow gains	23 ml/sec
Pressure gains	54 cm H_2_O
Breaths per minute (BPM)	30

The average breathing frequency was 40 breaths/min. Three semiautomatic tests, Boyle's law functional residual capacity (FRC) test, quasi‐static pressure volume (PV), and fast flow volume maneuver were measured using a forced pulmonary maneuver system (Buxco Electronics Inc., Sharon, CT) (Marcos et al., 2015; Radhakrishnan et al., 2015). Resistance and forced residual capacity (FRC) were determined with Boyle's law. Chord compliance (Cchord) was measured with a quasi‐static PV maneuver. Forced expiration volume in 400 ms (FEV400) and forced vital capacity (FVC) were recorded by the fast flow volume maneuver.

**FIGURE 2:**
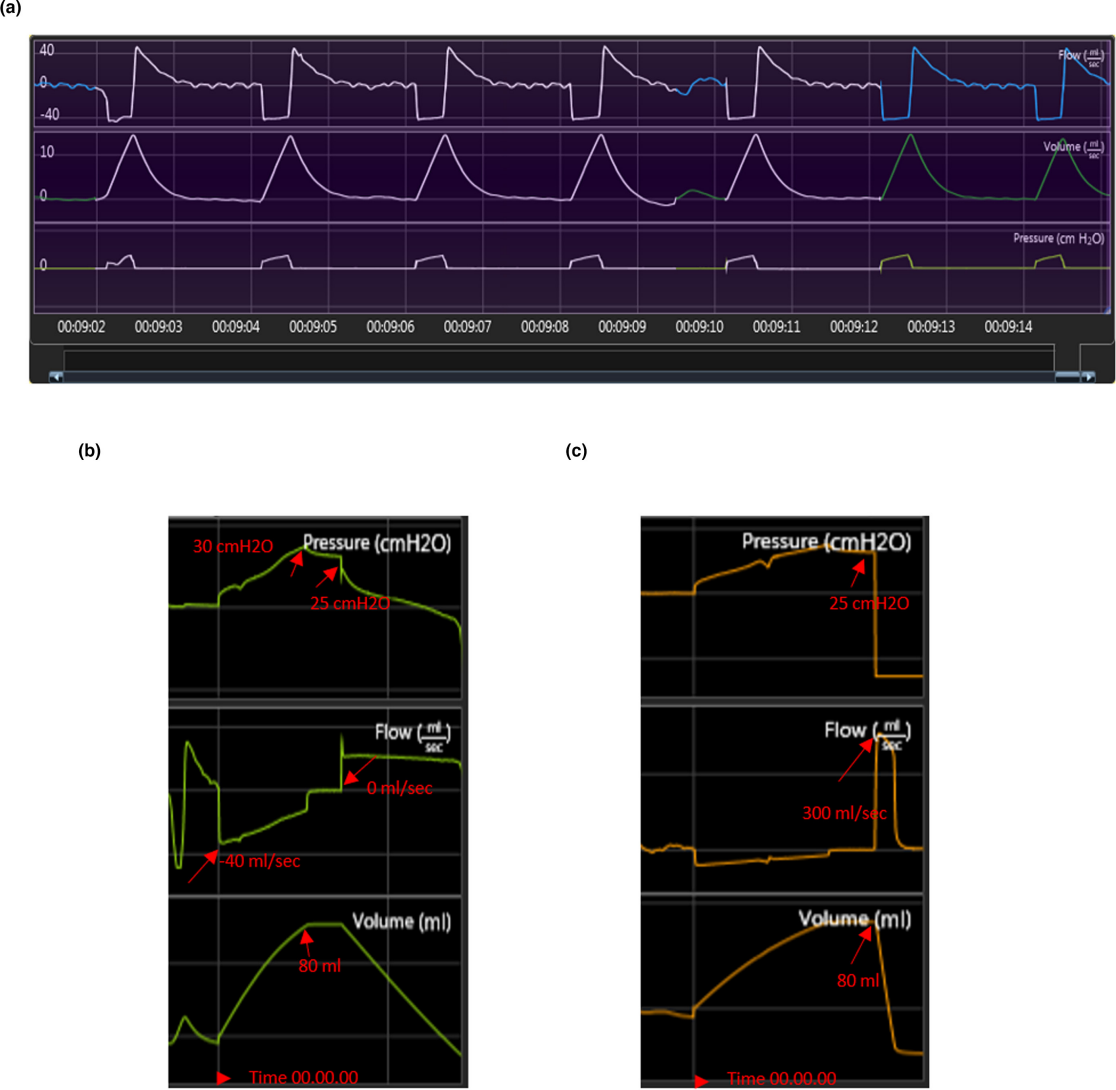
Lung function setting and parameters. (a) Normal breathing pattern of an anesthetized, orotracheally intubated, spontaneously breathing ferret. (b) Snapshot of pressure, flow, and volume parameters at the start of the PV test. (c) Snapshot of pressure, flow, and volume parameters at the start of the FV test.

Anesthetized animals were monitored for 2–3 mins and breathing patterns were recorded before the start of the PFT. First, breathing patterns were established for healthy sedated ferrets (Figure 2a). These baseline measures taken prior to LPS injury established the breathing patterns for each animal and decreased animal‐to‐animal variation. The FRC was measured using Boyle's law and defined the degree of airway occlusion at the end of expiration. Thus, the occlusion occurred when the pressure is 0 cm H_2_O, and the pressure should return to 0 in between efforts. The PV test provided information about the static lung properties, which are described by the pressure‐volume relationship of the lungs (supplemental methods). This test started when the flow is at 0 and the volume is at a minimum as shown in Figure 2b. The fast flow test provided information about the dynamic lung properties, which are described by the FV relationship of the lung. Since the pressure applied to the lung is essentially constant during the acquisition of the FV data, the flow value is proportional to the conductance of the limiting airways at a given expired volume (Figure 2c).

### 2.5 Bronchoalveolar lavage (BAL) collection post euthanasia

Post‐euthanasia, both lungs were removed from the thoracic cavity and tied to allow complete separation of the left and right lungs. An incision was made down the trachea leading to the tracheal bifurcation and a luer lock adapter was inserted in the right bronchus. A bolus of 20 ml of saline was then instilled into the left lung to fill all the lobes. With the syringe still connected in place, gentle squeezing of the lobes allowed for re‐collection of the infused lavage into the syringe. This was repeated twice for a total pooled volume of 40 ml lavage delivered to the lungs. Total cell counts were performed on the collected BAL samples, using trypan blue to measure viability. Cytospin slides of BAL samples were stained with a Differential Quick Stain Kit (modified Giemsa, Polysciences, Inc.) to obtain neutrophil counts.

### 2.6 Tissue collection and histology

Post‐euthanasia, ferret right lungs were collected for histology by slow inflation with 4% paraformaldehyde (PFA) solution followed by removal from the thoracic cavity. Lungs were immersed in 4% PFA solution for 48 h. After fixation, samples from each lung lobe (4 lobes total/animal) were cut, dehydrated in 70% ethanol, and embedded in paraffin. Tissue sections (5 μm) were cut, de‐paraffinized, and stained using Martius yellow–Crystal scarlet–Methyl blue (MSB) stain for fibrin and hematoxylin and eosin (H&E). Stained sections were scanned and analyzed using the Visiopharm software for quantification of the airspace/tissue ratio.

### 2.7 Pathology

H&E sections of ferret lungs (4 sections/animal, score, and averaged, *n* = 6 saline animals, *n* = 7 LPS animals) were examined by an American College of Veterinary Pathologists board‐certified pathologist in a blinded fashion. Scoring for inflammation was performed by counting the number of leukocytes in 10 consecutive alveoli at 400× magnification, choosing areas at random where the alveolar walls were intact. Finely fibrillary material, interpreted as fibrin present in the alveoli in the lung sections, was assessed as 0 = none, 1 = minimal, 2 = mild, 3 = moderate, and 4 = marked.

### 2.8 BCA protein assay

The total protein content of the BAL samples was measured using Pierce bicinchoninic acid (BCA) assay (Thermo Scientific). BAL samples were centrifuged at 500*g* for 5 mins at 4°C and the supernatant was collected and used to measure the total protein content at 562 nm absorbance. BAL protein content is expressed as mg/ml of BAL total volume collected.

### 2.9 Neutrophil elastase activity assay

Neutrophil elastase (NE) activity was assessed in the BAL samples using the Neutrophil Elastase Activity Assay Kit (Abcam). BAL samples were centrifuged at 500*g* for 5 mins and the supernatant was collected for this assay. NE activity was assessed based on the ability of NE in BAL samples to cleave a synthetic substrate that is added to the samples, thus releasing an AFC fluorophore which was quantified using a microplate reader at Ex/Em = 380/500 nm. Measurements were performed at 10 and 20 mins to assess the change in NE activity according to the following formula: ΔRFU = (RFU_20−_ RFU_20BG_) – (RFU_10−_ RFU_10BG_) where: RFU_20_ is the sample reading at 20 mins, RFU_20BG_ is the background sample at 20 mins, and RFU_10_ is the sample reading at 10 mins, RFU_10BG_ is the background sample at 10 mins. NE activity was calculated using the following formula:

NE activity = (B/V) × D = ng/ml, where B = amount of NE from the standard curve, V = original sample volume added into the reaction well (ml), and D = sample dilution factor.

### 2.10 TNF‐α levels

TNF‐α levels were measured in BAL samples using the Ferret TNF‐alpha/TNFA ELISA Pair Set (Sino Biological). BAL samples were centrifuged at 500*g* for 5 mins and the supernatant was collected for this assay. Briefly, ferret TNF‐alpha binds to an immobilized antibody that coats a 96‐well plate. The antibody–antigen–antibody sandwich is produced by adding horseradish peroxidase‐conjugated mouse anti‐ferret TNF‐alpha/TNFA ELISA pair monoclonal antibody. Optical density was then, measured using a microplate reader at 450 nm. Reported TNF‐α levels were reported in pg, after normalizing to the final volume of BAL samples for each animal.

### 2.11 Statistical analysis

Assays were performed with triplicate technical replicates, and data from the groups were pooled and displayed as mean ± standard error of the mean (SEM). Data were analyzed using GraphPad Prism version 7.0. An unpaired *t*‐test was used to determine statistical significance between the groups of saline and LPS. In lung function analysis, a mixed effects regression model was used to account for the paired nature of the data for each lung parameter. Post hoc pairwise comparisons were run on the group's least square means. *p*‐value <0.05 was considered significant.

## 3 RESULTS

### 3.1 Effect of LPS on lung function

**FIGURE 3:**
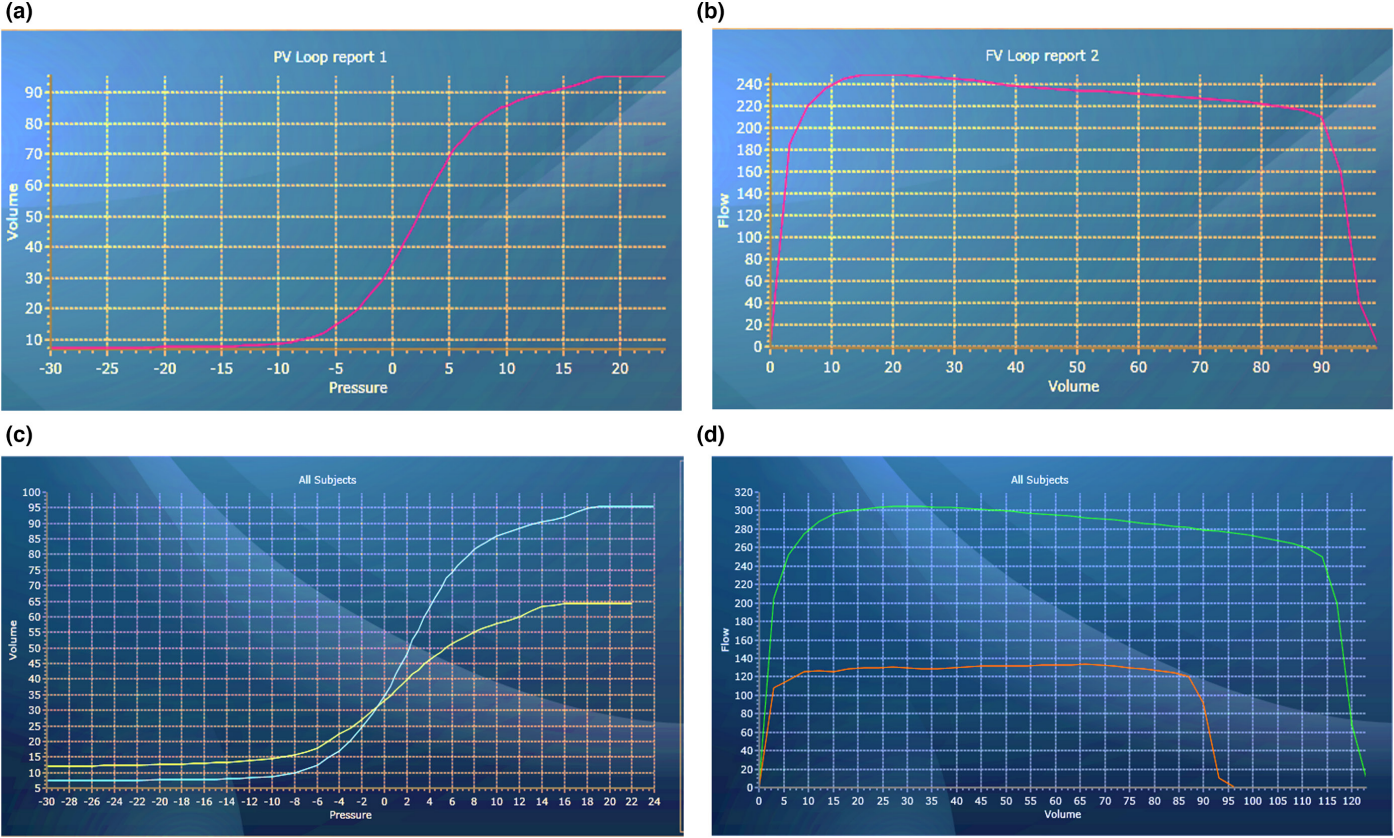
Lung function loop outputs. (a) Pressure‐Volume (PV) loop of healthy ferret lung. (b) Flow‐volume (FV) loop of healthy ferret lung. (c) PV loops during inspiration at baseline (blue line) and day 7 following LPS exposure (green line). (d) FV loops during inspiration at baseline (green line) and day 7 following LPS exposure (orange line).

**FIGURE 4:**
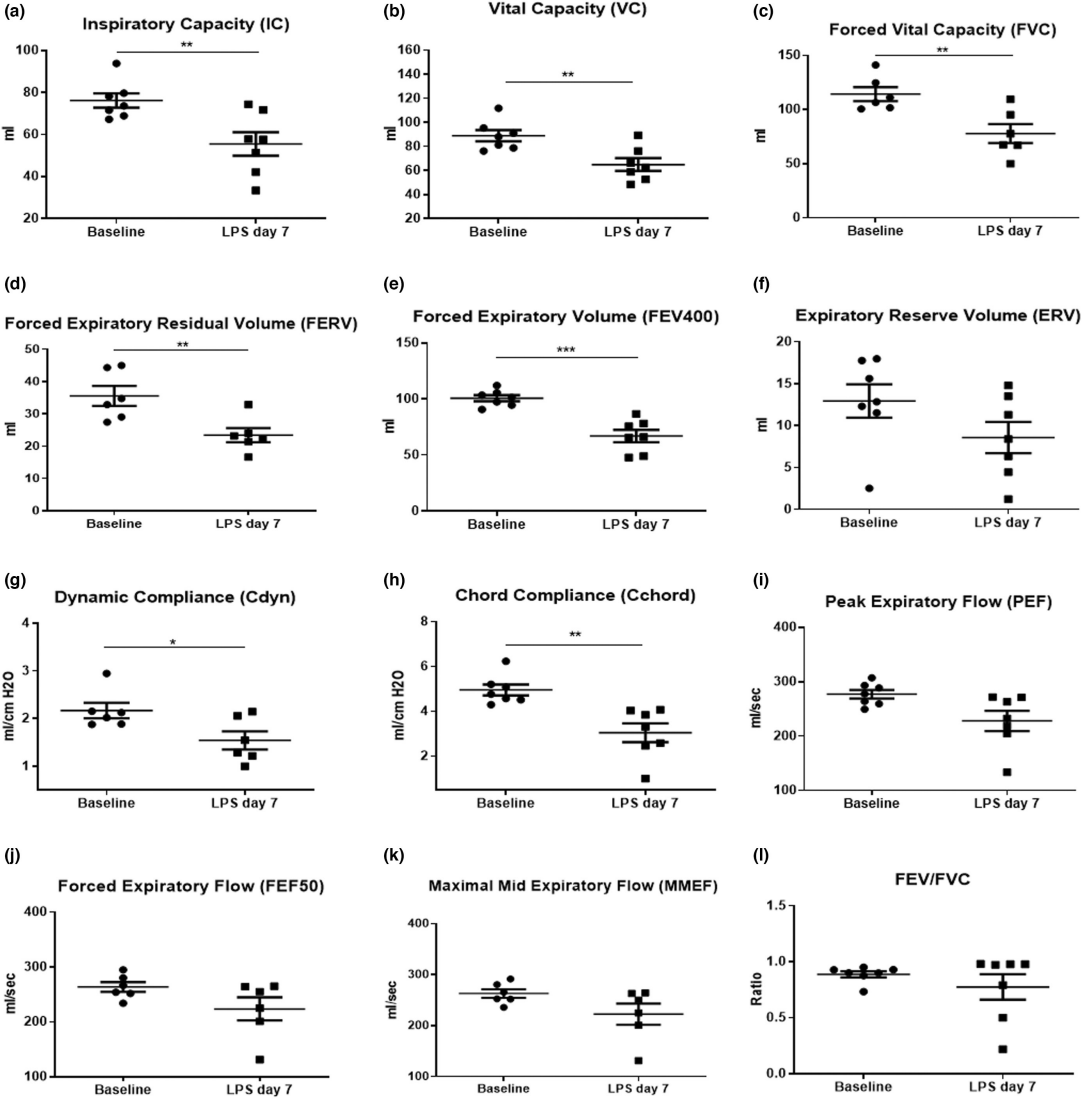
Lung function parameters measured at baseline and on day 7 following LPS administration. (a) Inspiratory Capacity (IC), (b) Vital Capacity (VC), (c) Forced Vital Capacity (FVC), (d) Forced Expiratory Residual Volume (FERV), (e) Forced Expiratory Volume at 400 ms (FEV400), (f) Expiratory Reserve Volume (ERV), (g) Dynamic Compliance (Cdyn), (h) Chord Compliance (Cchord), (i) Peak Expiratory Flow (PEF), (j) Forced Expiratory Flow (FEF50), (k) Maximal Mid Expiratory Flow (MMEF), (l) FEV/FVC ratio. *n* = 7 LPS. Bars represent SEM. *p‐value < 0.05. **p‐value < 0.01. ***p‐value < 0.001.

Ferrets underwent PFT before any LPS administration to establish baseline measurements for each animal. We measured the PV (Figure 3a) and FV (Figure 3b) loops in healthy ferrets at baseline. The slope of the PV curve determines the instantaneous compliance at a given pressure. On day 7, PFT was performed again to measure the effect of LPS exposure. Compared to baseline, PFT generated in animals after 7 days of LPS exposure exhibited a decrease in volume (Figure 3b) and flow (Figure 3c), characteristics of restrictive pulmonary disorders. Specifically, administering LPS decreased inspiratory capacity (IC) (75.9 ± 10.2 ml vs. 55.5 ± 14.8 ml, *p* < 0.01), vital capacity (VC) (90 ± 10.7 ml vs. 64.8 ± 14 ml, *p* < 0.01) and FVC (112.6 ± 16.1 ml vs. 77.8 ± 21.4 ml, *p* < 0.01) by day 7 of LPS administration compared to the baseline (Figure 4a–c). Furthermore, we also observed decreased forced expiratory residual volume (FERV) (35.2 ± 6.6 ml vs. 23.3 ± 5.3 ml, *p* < 0.01) and lower FEV400 (98.6 ± 13.1 ml vs. 66.9 ± 14.6 ml, *p* < 0.001) at day 7 post LPS compared to baseline measurements. Although the expiratory reserve volume (ERV) decreased following LPS, changes were not statistically significant (Figure 4d–f). Compared to baseline, ferrets receiving LPS also exhibited lower dynamic compliance (Cdyn) (2.1 ± 0.4 ml/cm H_2_O vs. 1.5 ± 0.5 ml/cm H_2_O, *p* < 0.05) and cChord (5 ± 0.6 ml/cm H_2_O vs. 3 ± 1.1 ml/cm H_2_O, *p* < 0.01) (Figure 4g,h). LPS administration decreased flow parameters such as peak expiratory flow (PEF), forced expiratory flow (FEF50), and maximal mid expiratory flow (MMEF), however, changes were not statistically significant (Figure 4i–l) from baseline values. In addition, the FEV/FVC ratio remained unchanged after LPS administration. Animals receiving saline only did not show any significant alterations to PFT parameters, except for increased dynamic compliance (Cdyn) (Figure S1). We do not yet understand the reason for this observed increase in our model. In the clinical setting, static compliance is the preferred compliance metric, with dynamic compliance considered unreliable. Therefore, we would suggest that dynamic compliance should not be considered a useful metric for studying lung function in this model.

### 3.2 Effect of LPS on immune cell infiltration and inflammation

**FIGURE 5:**
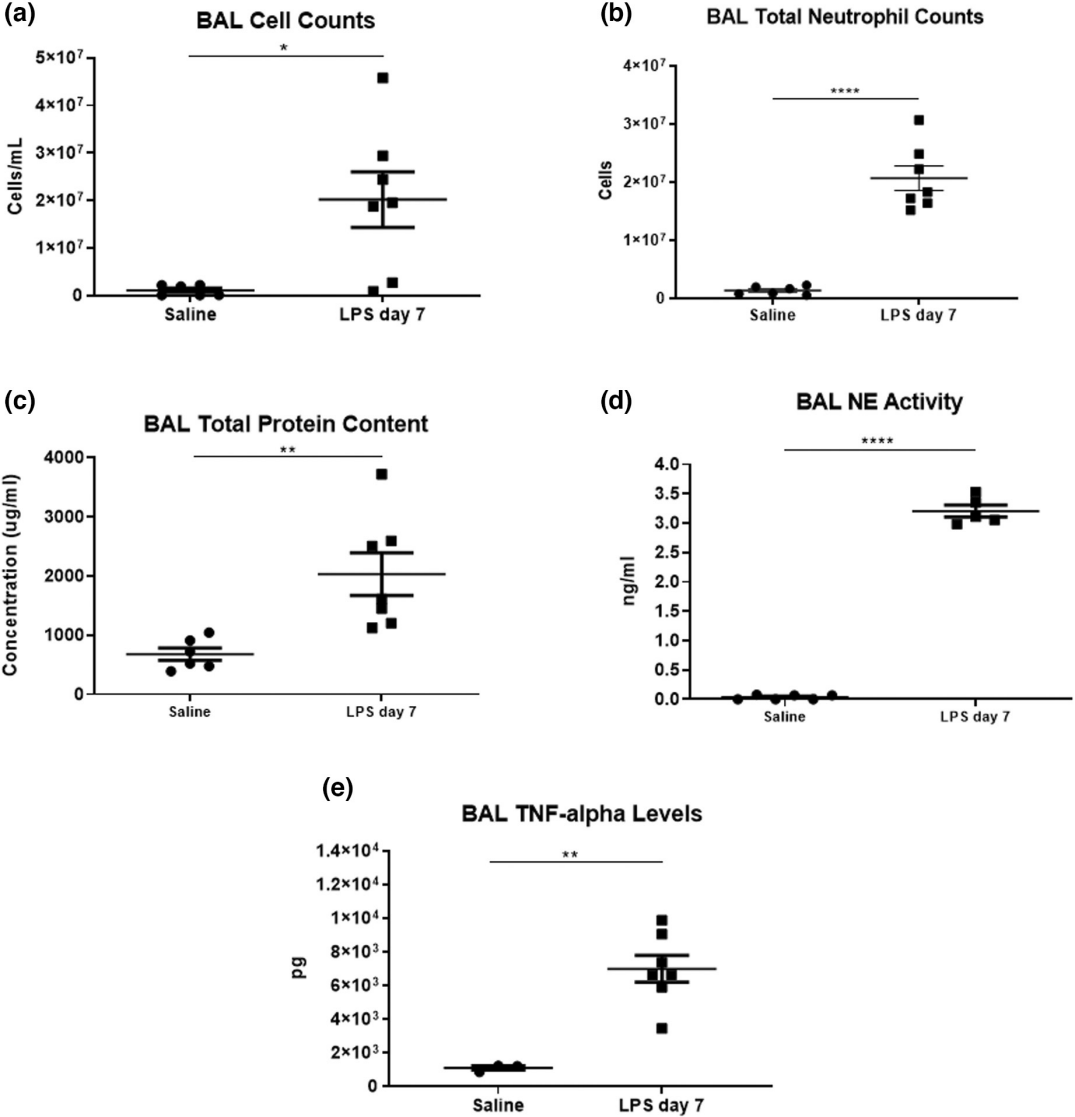
Analysis of bronchoalveolar lavage (BAL) cell counts and protein levels for samples collected following saline and LPS administration at day 7. (a) BAL total counts, (b) BAL total neutrophil counts, (c) BAL total protein content, (d) BAL NE activity. (e) BAL TNF‐α levels. *n* = 6 saline (*n* = 3 saline for TNF‐α), *n* = 7 LPS. Bars represent SEM. *p‐value < 0.05. **p‐value < 0.01, ****p‐value < 0.0001.

Ferrets that received LPS exhibited a higher number of total cells counts performed on BAL samples compared to saline controls (2.3 × 10^7^ ± 1.4 × 10^7^ cells vs 1 × 10^6^ ± 1.3 × 10^4^ cells, *p* < 0.05) (Figure 5a). Total neutrophil counts showed that LPS administration significantly increased neutrophils in BALs, (2 × 10^7^ ± 5.5 × 10^6^ cells vs. 1.36 × 10^6^ ± 7 × 10^5^ cells *p* < 0.0001) compared to saline controls (Figure 5b). BAL total protein content was also elevated in LPS‐treated animals when compared to saline animals (2 × 10^3^ ± 0.9 × 10^3^ vs. 0.6 × 10^3^ ± 0.2 × 10^3^ mg/ml, *p* < 0.01). Similarly, LPS ferrets had higher NE activity in their BALs compared to saline controls (0.03 ± 0.03 vs. 3.2 ± 0.22 ng/ml, *p* < 0.0001) (Figure 5c,d). In addition, levels of TNF‐alpha measured in BAL samples showed that LPS ferrets exhibit a significantly higher level of this inflammatory marker in BAL samples compared to saline controls (6.9 × 10^3^ ± 2 × 10^3^ vs. 1 × 10^3^ ± 0.2 × 10^3^ pg, *p* < 0.01) (Figure 5e).

### 3.3 Effect of LPS on pulmonary tissue structure

**FIGURE 6:**
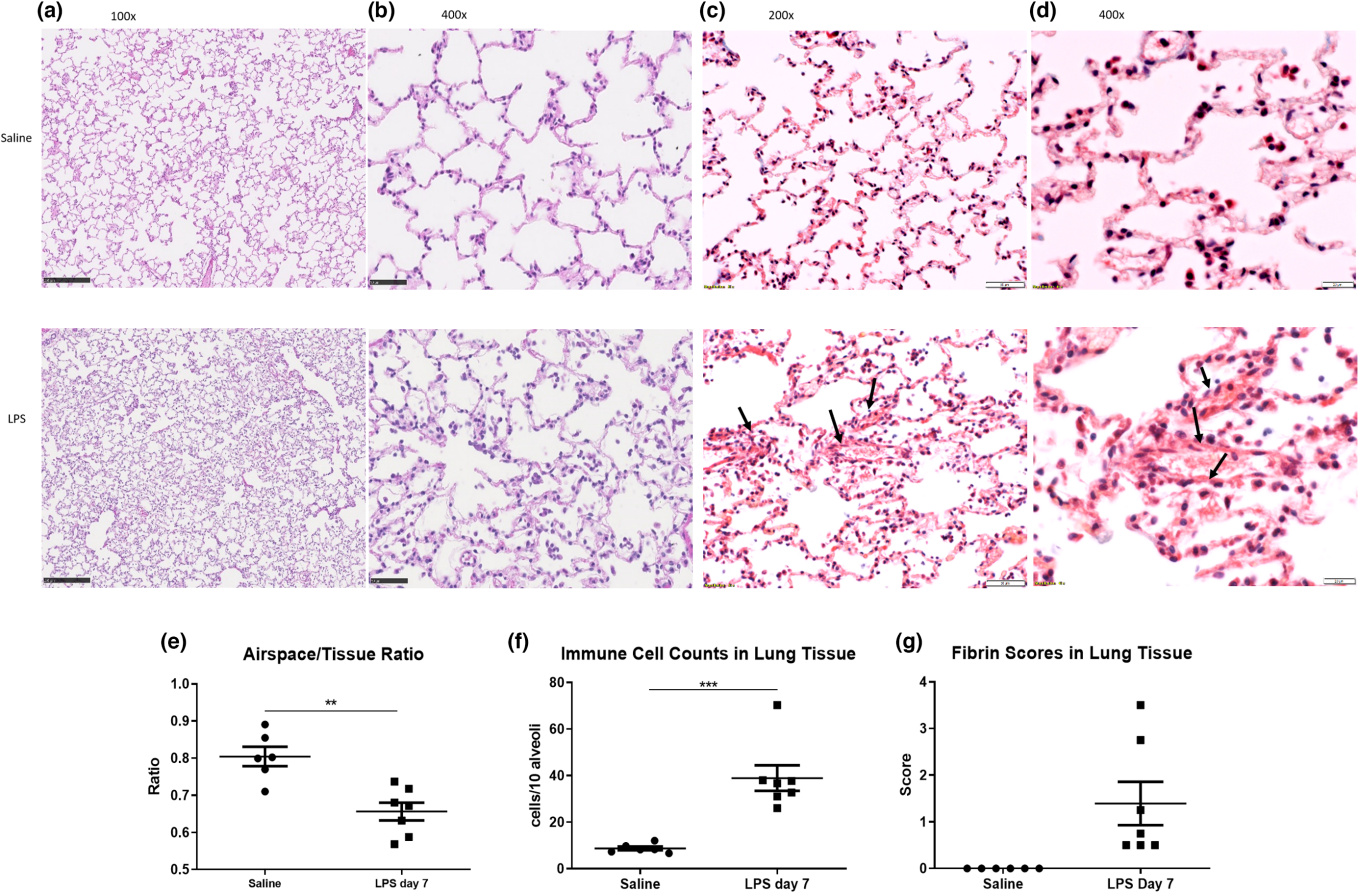
Lung histological changes in ferrets following saline and/or LPS administration. (a) Representative photomicrographs of pulmonary mesenchyme and alveolar spaces in H&E stained sections at 100× magnification, (b) at 400× magnification, (c) Representative photomicrographs of “fibrin balls” (arrows) in the alveolar space at 200× magnification, (d) at 400× magnification (e) Airspace/Tissue ratio. (f) Immune cell counts in lung tissue. (g) Fibrin score in lung tissue. *n* = 6 for saline, *n* = 7 LPS. Bars represent SEM. *p‐value < 0.05. **p‐value < 0.01. ***p‐value < 0.001.

Representative images of lung tissue stained with H&E (*n* = 4 sections/animal, 13 animals total) showed distortion of alveolar structures in ferrets that received LPS when compared to saline animals (Figure 6a,b). MSB stain for fibrin revealed patchy intra‐alveolar fibrin deposition in the form of “fibrin balls” in lung tissue obtained from LPS ferrets. (Figure 6c,d). Quantitative analysis of H&E sections showed that ferrets exposed to LPS had a decrease in airspace/tissue ratio (0.8 ± 0.06 vs. 0.66 ± 0.06, *p* < 0.01) (Figure 6e) consistent with the observed thickening of the alveolar linings after LPS treatment when compared to saline animals. In addition, tissue sections showed the presence of a higher number of immune cells in the alveolar and airway spaces in LPS when compared to the saline group (8.7 ± 1.9 cells/10 alveoli vs. 38.9 ± 14 cells/10 alveoli, *p* < 0.001) (Figure 6f). Finally, a blinded pathological evaluation of the ferret section performed by an independent pathologist showed that LPS ferrets exhibited a mild increase in fibrin deposition in pulmonary tissue, confirming that LPS administration caused an inflammatory response in ferret lung tissue (Figure 6g).

## 4 DISCUSSION

A key objective of this study was to develop a pre‐clinical animal model that recapitulates aspects of the pathophysiology of inflammatory lung diseases.

In lung diseases, lung function testing is an important parameter to determine, as it is a robust indication of the type and severity of the disease. To our knowledge, this is the first study to use forced maneuver lung function testing on live ferrets. In this study, we performed a pre and post‐injury lung function evaluation of lung parameters to first, establish baseline lung function in healthy live ferrets and second, to evaluate the effect of LPS administration on lung function. Our approach to perform PFT using multiple forced maneuvers system on live ferrets generated findings that are similar to data obtained from human patients undergoing lung function testing in the clinic.

One of the main advantages of a ferret lung injury model is its lung biology and physiology that is similar to humans. Ferrets have portions of the upper and lower respiratory tracts, as well the number of generations of terminal bronchioles, and expression of relevant receptors and mutations (such as influenza receptor and CFTR mutation) that are similar to humans, resulting in similar disease pathogenesis (Johnson‐Delaney & Orosz, 2011; Leigh et al., 1986; Li & Engelhardt, 2003; Munoz‐Fontela et al., 2020; Oldham et al., 1990; Plopper et al., 1980). For this reason, ferrets have been used extensively as models for human respiratory diseases such as cystic fibrosis and viral infections like influenza and SARS‐COV‐2. Another important advantage of the ferret model is the ability to perform repeated intubations required for longitudinal PFT studies. While PFT is regularly performed in smaller animals such as mice, repeated intubations in small animals are challenging and risks detrimental impacts on PFT data collection. Ferrets are easily intubated with minimal manipulation, and the use of an inflatable cuff intubation tube provides reproducible and consistent data with minimal impact on the airway. Finally, ferrets are a cost‐effective alternative to larger animals such as dogs, pigs, and non‐human primates with regard to animal housing and maintenance (Enkirch & von Messling, 2015). For example, ferrets are approximately 10× cheaper than non‐human primates to purchase, as well as being significantly easier to procure and ship. Additionally, per diem rates for ferrets are on the order of 4 times cheaper than dogs and pigs. Therefore, ferrets represent a useful compromise between versatility and costs that have significant advantages for studies of pulmonary injury, disease, and function.

We measured baseline and post‐LPS lung function in the same animal, as such each animal served as its own control. Determining the baseline measures for each animal is important to accurately evaluate the severity of the LPS‐induced pulmonary injury. This process is in line with clinical PFT procedures, where each patients' PFTs are compared longitudinally to determine worsening or improvement of disease and/or treatment. Thus, we were able to accurately report the change in lung function after LPS injury compared to the pre‐LPS lung function baseline. Performing pre‐ and post‐LPS PFTs permitted the use of the mixed effect regression statistical analysis that takes the pairwise baseline and post‐injury data for each animal into account while statistically comparing the entire cohort. By doing so, even when lung function parameters are different among the animals due to the normal expected animal variability, we were able to detect the consistent decrease in lung function caused by LPS. Without knowing what the baseline is for each animal, we would not determine the true effect of LPS by just comparing it to entirely different control animals. Similarly, in human patients, PFTs are compared to the patient's previous admissions to determine disease progression, treatment options, and drug effects.

Lung injury was significant on day 7 post LPS in which ferrets had a decline in FVC and a 30% drop in their FEV400 compared to baseline. The animals experienced shorter but more frequent breaths, indicating a decline in pulmonary airflow. The FEV/FVC ratio determines the amount of air that can be exhaled during a certain time (in ferrets this is adopted at 400 ms, hence FEV400), versus the total amount of air that can be exhaled (Mannino et al., 2003; Miki et al., 2016). As observed in our data analysis, the ratio of FEV400/FVC remained proportional throughout our studies, which is consistent with a restrictive lung injury. Although differences were not statistically significant, ferrets did show decreased PEF and MMEF at day 7 when compared to baseline measurements. The potential decrease in these flow parameters indicates that LPS administration could cause pathology in the smaller as well as in the larger airways (Kawakami et al., 1990; Marseglia et al., 2007). The decline in compliance parameters Cchord and Cdyn, which determine the dispensability and elasticity of lung tissue, suggests that the lungs exhibit more stiffness and have limited expansion. The observed decline in lung function following LPS administration coincides with a similar decline in PFT patterns observed in humans patients with ALI (Johnson & Theurer, 2014; Schelling et al., 2000). Oxygen saturation (SpO_2_) and weight monitoring were performed throughout the study, where SpO_2_ remained at 99%, and weights did not significantly change from baseline in all groups. The absence of significant difference in these parameters further supports a mild ALI injury. Thus, the administration of LPS in ferrets was able to recapitulate the lung function aspects observed in the human population in a clinically relevant manner (Arnett, 1935).

We also characterized the inflammatory response elicited by LPS, which was evidenced by increased protein levels and immune cell counts in BAL samples. Human patients demonstrate increased immune cell infiltration into their lungs, edema, and increased vascular leakage due to the high levels of inflammatory markers (Dushianthan et al., 2011; Menezes et al., 1985). The targeted delivery of LPS into the lungs of ferrets accomplished by nebulization developed a localized inflammatory response that is similar to the one observed in human patients (Abraham, 2003; Goodman et al., 2003). Moreover, persistently high levels of neutrophils in BALs after the first week of inflammatory lung injury have been linked to disease severity and higher mortality rates (Grommes & Soehnlein, 2011; Lee & Downey, 2001; Steinberg et al., 1994; Welbourn & Young, 1992). In our model, we were successful in recapitulating the persistent increase in neutrophil counts by administering daily repeated doses of LPS. This inflammatory response was ongoing in ferret lungs by day 7 whereby high numbers of neutrophils and associated NE levels were detected. Elevated levels of NE in BALs are associated with extracellular matrix degradation and thus could serve as a potential contributor to the tissue loss of airspace/tissue space we started to observe on day 7 (Polverino et al., 2017). TNF‐α levels were significantly elevated in the BALs of LPS ferrets, further validating the activation of the immune response by LPS. The sources of TNF‐α could well be activated macrophages in BALs. This finding is in accordance with many other in vivo studies that show increased TNF‐α levels following LPS, which contributes to the exacerbation of the inflammatory response, and disease progression especially in lung disorders (Goto et al., 2004; Michie et al., 1988; Mukhopadhyay et al., 2006; Niederman & Fein, 1990; Sheridan et al., 1997). It is important to note that the LPS exposure endpoint at day 7 paralleled the inflammatory response and decline in lung function, thus addressing our aim to develop a model that closely represents human lung inflammation (Johnson & Matthay, 2010).

The downstream effect of lung injury is tissue damage that could arise especially in patients developing more severe adverse conditions (Matthay & Zemans, 2011). This ferret model successfully recapitulated the acute inflammatory response that occurs in these diseases, as it partially represented the subsequent tissue damage and fibrosis that occurs. Even though we observed an increase in tissue/airspace ratio by day 7, along with increased fibrin deposition in lung tissue, the 7‐day duration of LPS exposure may be too early to show major histological changes as tissue damage requires a prolonged period of injury time (Fein & Calalang‐Colucci, 2000; Fukuda et al., 1987). Nonetheless, the presence of immune cells in alveolar and airway spaces and the histological changes that were detected, indicate that the LPS caused tissue injury as early as 7 days. Prolonged LPS exposure beyond 7 days could lead to the development of more severe tissue damage.

Our study shows a successful induction of lung injury through LPS administration, showing accurate physiological, functional, and histological changes that are consistent with clinical manifestations of lung diseases in human patients. In future studies, we aim to use CF, viral, and/or bacterial infected animals to increase the model's disease specificity and impact.

This study also highlights the advantages of the use of a larger animal including: performing multiple PFTs per animal, thus accurately defining baseline lung function for each animal, closely recapitulating clinical measurements, and generating accurate comparisons of post‐injury outcomes. Our study provides a model of lung injury that can be an improvement on the existing models, offering the possibility of performing repeated PFT measurements with minimal invasiveness and a highly precise clinically relevant approach. By accurately modeling the acute phase of ALI, our ferret model constitutes a useful platform to test new therapies for lung diseases such as CF. Transgenic CF ferrets are valuable models to study the onset and progression of CF because ferrets exhibit lung pathology, airway obstruction, and accumulation of thick mucus similarly to human patients (Cho et al., 2010; Keiser et al., 2015; Sun et al., 2008, 2014; Yan et al., 2015). However, adult CF ferrets do not exhibit any immune response dysfunction as human patients (Semaniakou et al., 2018) so our model can be used to develop specific disease pathogenesis such as viral and bacterial CF models.

### AUTHOR CONTRIBUTION

Study conception and design: Oula Khoury, Cara Clouse, Sean V. Murphy, Jeffrey Applegate, Anthony Atala; Data collection: Oula Khoury, Cara Clouse, Malcolm K. McSwain, Nancy Kock, Sean V. Murphy; Analysis and interpretation of results: Oula Khoury, Sean V. Murphy; Draft manuscript preparation: Oula Khoury. All authors reviewed the results and approved the final version of the manuscript.

## FUNDING INFORMATION

This work was supported by the Lisa Dean Moseley Foundation Stem Cell Research Grant.

## CONFLICT OF INTEREST

None of the authors have any conflict of interest to disclose.

## ETHICS STATEMENT

All animal procedures were performed according to the protocols approved by the Wake Forest University Institutional Animal Care and Use Committee (A20‐105 and A21‐062). All experiments were performed in accordance with Animal Care and Use Committee guidelines and regulations.

## Supporting information


Figure S1

Appendix S1
