# Supplementary figures and images for: Ferret acute lung injury model induced by repeated nebulized lipopolysaccharide administration
Source: Physiol Rep. 2022 Oct 21;10(20):e15400. doi: 10.14814/phy2.15400 (PMC9585421; doi:10.14814/phy2.15400)

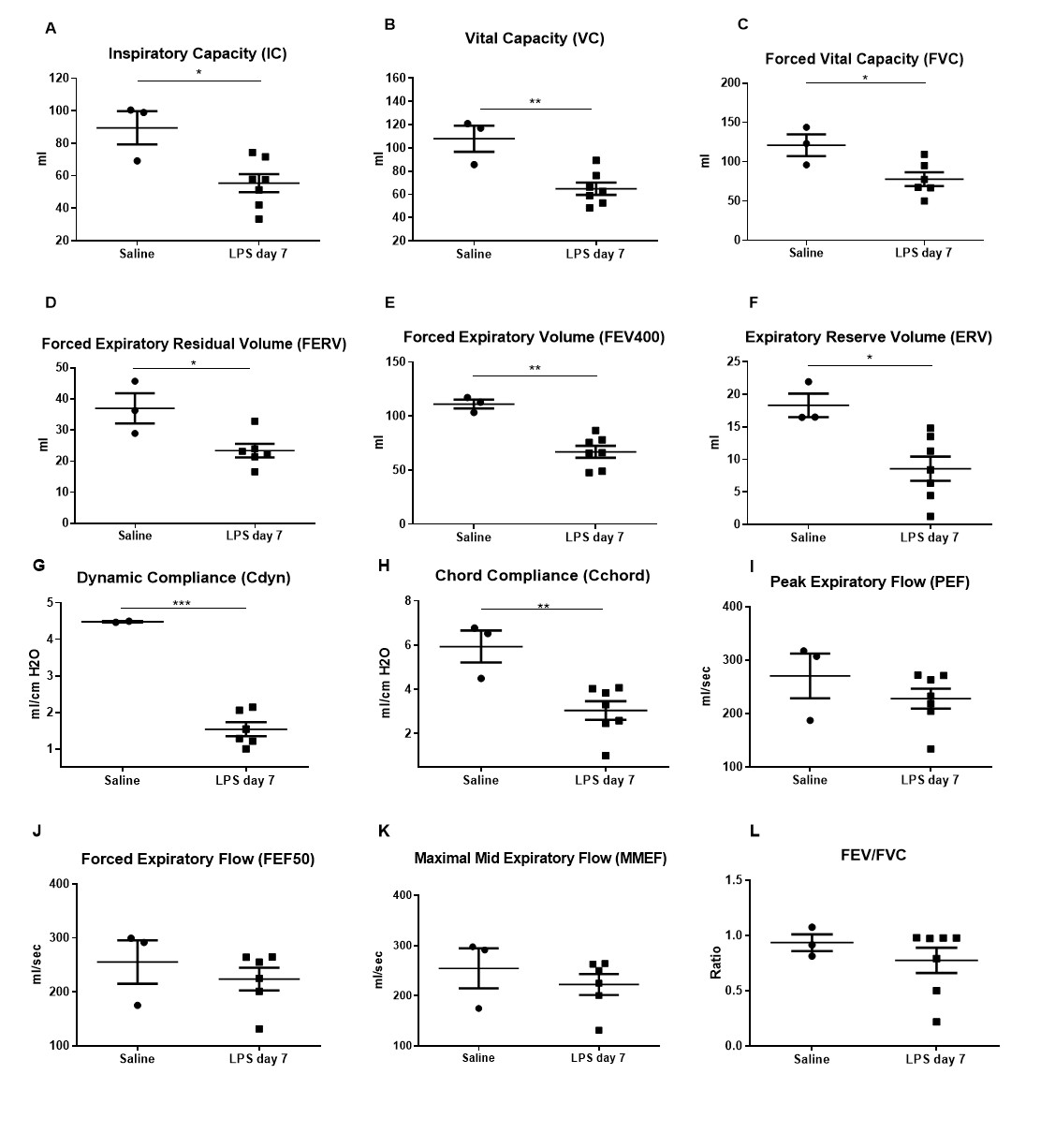

Supplement: Supplementary file 1 — Figure S1 [file PHY2-10-e15400-s001.jpg]
