# Supplementary material for: Ferret acute lung injury model induced by repeated nebulized lipopolysaccharide administration
Source: Physiol Rep. 2022 Oct 21;10(20):e15400. doi: 10.14814/phy2.15400 (PMC9585421; doi:10.14814/phy2.15400)
Supplement: Supplementary file 2 — Appendix S1 [file PHY2-10-e15400-s002.docx]

Supplemental Methods:

Animal Sedation

Animals were weighed and a baseline physical examination was performed. The animals were sedated with an incremental increase in isoflurane inhalant that was administered via an 18.5 L anesthesia chamber (Patterson Veterinary, Greeley, CO). Once the animal showed no response to stimuli, it was then maintained via mask. Anesthetic levels were managed based on vital parameters. An intravenous 24g catheter (Terumo, Tokyo, Japan) was placed in either the right or left cephalic veins. The animal was then intubated using a 2.5 mm ID, cuffed endotracheal tube that was placed to the level of the thoracic inlet (Teleflex, Wayne, PA). The pilot balloon was inflated to ensure there were no leaks around the tube. Ensuring that the animal was anesthetically stable via pulse-oximetry, a slow bolus of injectable anesthetic was provided via the IV catheter. This cocktail consisted of ketamine at 40 mg/kg (Vedco, Saint Josef, MO), xylazine at 1 mg/kg (Akorn Animal Health, Lake Forest, IL), and buprenorphine at 0.003 mg/kg (Par Pharmaceutical, Woodcliff Lake, NJ). The administration of 0.1- 0.15 ml of this cocktail showed a slow decline of 10-20 beats per minute (bpm) in heart rate and minimal to no decrease in SpO_2_. This provided approximately 15-20 minutes of a surgical anesthesia. In parallel, the isoflurane level was reduced to zero and the breathing circuit flushed with O_2_ after initiating the injection of the anesthetic. Continued anesthetic monitoring included mucous membrane color and response to stimuli (from the pulmonary function testing (PFT) apparatus). Additional anesthetic bolus could be provided in 0.05 ml increments of the cocktail or ketamine alone via the IV catheter as necessary based on response to stimuli. Once the PFT procedure was completed, the animal was removed from the apparatus. Pulse-oximetry monitoring and heat supplementation were replaced. Generally, mild comparative tachycardia and decreased SpO_2_ (80-90%) was observed directly post PFT.

Lung Function Testing

The PV relation describes the mechanical behavior of respiratory system at different levels of lung inflation and deflation. Thus, the PV curve has been used to determine disease severity and measure reduction in lung compliance. In our pulmonary testing system, the PV curve was obtained by slowly inflating the chest, either continuously or in a series of small steps. The PV is described as a true sigmoid curve. The first segment is characterized by low compliance. The intermediate segment, which falls between 2 points, the upper and lower inflection points, can be considered linear and is used to measure the 'linear' compliance. Beyond the upper inflection point, the PV curve tends to flatten again (1). The inflection points delineate the levels of opening and closing pressures and thus constitute useful markers to determine pulmonary abnormalities

1. Brochard L. What is a pressure-volume curve? Critical care. 2006;10(4):156.
